# Evaluation of Bronchoscopy and Bronchoalveolar Lavage Findings in Cats With *Aelurostrongylus abstrusus* in Comparison to Cats With Feline Bronchial Disease

**DOI:** 10.3389/fvets.2019.00337

**Published:** 2019-10-02

**Authors:** Paolo E. Crisi, Lynelle R. Johnson, Angela Di Cesare, Francesca De Santis, Morena Di Tommaso, Simone Morelli, Stefania Pantaleo, Alessia Luciani, Roland Schaper, Fabrizio Pampurini, Andrea Boari

**Affiliations:** ^1^Faculty of Veterinary Medicine, University Teaching Veterinary Hospital, University of Teramo, Teramo, Italy; ^2^Department of Medicine and Epidemiology, University of California, Davis, Davis, CA, United States; ^3^Bayer Animal Health GmbH, Leverkusen, Germany; ^4^Bayer Animal Health, Milan, Italy

**Keywords:** aelurostrongylosis, feline bronchial disease, bronchoschopy, bronchoalveolar lavage, bronchiectasis

## Abstract

The cat lungworm *Aelurostrongylus abstrusus* is a cause of lower respiratory tract disease worldwide. Bronchoscopy and bronchoalveolar lavage (BAL) are important tools for diagnosing respiratory diseases in cats. Therefore, the aim of the study was to investigate the usefulness of bronchoscopy and BAL in the diagnosis of *A. abstrusus*. Findings from bronchoscopic examination and BAL of 24 naturally infected cats were evaluated and compared with those of 12 cats with idiopathic Feline Bronchial Diseases (FBDs). Data were analyzed using Mann-Whitney or Fisher's exact tests. No significant bronchoscopic differences were detected between cats with aelurostrongylosis and FBDs in bronchial mucus, nodular lesions, and airway collapse. On the other hand, airway hyperemia, epithelial irregularities, and bronchial stenosis were observed more frequently in cats affected by FBDs than aelurostrongylosis, while bronchiectasis was found only in cats infected by *A. abstrusus*. Neutrophilic, eosinophilic, lymphocytic, and mixed inflammation were recorded in both groups. Bacteria or bacterial DNA was identified regardless of the presence or absence of *A. abstrusus* with no significant differences between groups. Larvae of *A. abstrusus* were cytologically detected in 5 of the 24 cats (20.8%) with aelurostrongylosis. These results indicate that, although some findings on bronchoscopic examination (i.e., bronchiectasis) can be described more frequently in cats infected by *A. abstrusus*, bronchial alterations and cytological findings in aelurostrongylosis are not specific unless larvae are observed and overlap with those of other feline airway diseases.

## Introduction

Cats affected by lower respiratory tract disease often present for a chronic cough with gradually increasing respiratory distress and non-specific signs such as exercise intolerance and anorexia. Common causes include inflammatory, infectious, or parasitic diseases (1, 2). *Aelurostrongylus abstrusus* is the most important nematode affecting the respiratory system of domestic cats (*Felis silvestris catus*) (3). This parasite occurs worldwide and, in areas where it is endemic, aelurostrongyslosis represents one of the main differential diagnoses for lower respiratory tract disease in cats (3).

Copromicroscopic examination is a simple, inexpensive and reliable in-house test, and should always be considered as a first step in the diagnostic work-up for feline lower airway diseases, especially in at-risk subjects (4). The Baermann test is considered the gold standard to diagnose feline aelurostrongylosis (3). However, false negative results are possible, it may be necessary to leave the specimen set up overnight and repeated examinations are necessary to enhance its sensitivity, thus, in some cases, it may take several days.

Bronchoscopy is one of the most useful techniques for providing a diagnosis in small animals with airway or lung disease, however its role in diagnosis of feline aelurostrongylosis is unclear. In particular, bronchoscopic evaluation allows visualization of airway abnormalities and collection of bronchoalveolar lavage (BAL) samples for the diagnosis of many respiratory diseases. Moreover, the cytological and microbiological evaluation of BAL fluid can be used as an auxiliary tool to characterize lower airway disorders and antimicrobial sensitivity can be used to determine antibiotic susceptibility in treating bacterial infections (5–7).

The aims of this study were to investigate endoscopic and BAL fluid features of *A. abstrusus*-infected cats and to determine their usefulness in the diagnosis of feline aelurostrongylosis in comparison to findings recorded in cats with idiopathic Feline Bronchial Diseases (FBDs).

## Materials and Methods

### Animal Selection and Study Design

Between April 2016 to June 2018, 36 privately owned cats, were examined at the Veterinary Teaching Hospital (VTH) of the Faculty of Veterinary Medicine of Teramo, Italy (*n* = 25) and at the William R. Pritchard Veterinary Medical Teaching Hospital, University of California, Davis, USA (*n* = 11).

All cats were referred for lower respiratory tract signs and underwent complete clinical examination, with a thorough history, complete cell blood count, serum chemistry profile. The retroviral status (SNAP FIV/FeLV Combo Test, IDEXX Laboratories) and two or three orthogonal thoracic radiographs were also obtained.

Twenty-four cats were infected by *A. abstrusus* (Group Aa) and the diagnosis of infection was made by the fecal Baermann test and confirmed with PCRs specific for cat metastrongyloids (8, 9). First stage larvae (L1s) found at the Baermann examination were identified based on key morphometric (i.e., length 360–415 μm) and morphological (i.e., pointed head with subterminal-dorsal oral opening, S-shaped tail with knob-like or small finger-like projections at tip of cuticular spines) characteristics (3, 10). In order to estimate the parasitic load in terms of larvae per gram of feces (LPG), study cats were subjected to quantitative Baermann test.

Twelve cats diagnosed with idiopathic FBDs were enrolled as control group (Group FBDs).

Eligible cats were examined by bronchoscopy with BAL, and BAL cytology and microbiology. All study cats were privately owned naturally infected cats for which each owner had signed a consent form and agreed to participate in the study. The study was approved by the Committee on Animal Research and Ethics at the Universities of Chieti-Pescara, Teramo, and Experimental Zooprophylactic Institute of AeM (CEISA), protocol UNICHD12 n. 1773 title VI, 8.

### Bronchoscopy and BAL Examinations

Animals were pre-medicated with butorphanol (0.2 mg/kg IM), or methadone (0.2 mg/kg IM) and ketamine (2 mg/kg IM), or dexmedetomidine (1 μg/kg IM) and ketamine (2 mg/kg IM), and anesthesia was induced with propofol (2–4 mg/kg IV) and midazolam (0.2 mg/Kg IV). After anesthetic induction, topical spray of aerosolized 2% lidocaine was administered to reduce the risk of laryngospasm, if necessary. Maintenance of anesthesia was obtained with intermittent bolus of propofol as needed or with a constant rate of infusion. Endoscopy was performed in sternal recumbency with a flexible videoendoscope (Olympus^®^ BIF 3C160, Melville, NY, USA) with an insertion tube outer diameter of 3.8 mm and a 1.2 mm channel. During endoscopy the following findings were evaluated and recorded: excessive bronchial mucus, airway hyperemia, epithelial irregularities or nodular lesions, airway collapse (defined as a flattening of the airways) at rest or with suction during BAL, stenosis of airway openings (recognized as circumferential narrowing of the airway that precluded bronchoscopic interrogation of more distal airways), and bronchiectasis (7). Investigators (PEC and LRJ) at different institutions agreed on bronchoscopic methodology and interpretation of findings on visual inspection, using a detailed procedure description as an interpretative guide (11).

BAL was performed as previously described (11), with a 3–10 ml aliquot of warm sterile saline solution instilled at the bronchial segment RB2 or, when possible, at two distal segments (12, 13).

Cytology was performed on cytospin samples of BAL stained with May-Grunwald-Giemsa. Differential cell counts were classified in accordance with the predominant inflammatory cell type as neutrophilic, lymphocytic, eosinophilic, or mixed, as described previously (13). An aliquot of sterile BAL was subjected to bacterial culture on Plate Count Agar (PCA). The diagnostic threshold for semi-quantitative cultures of BAL fluid was 104 cfu/ml and bacteria from pure cultures were identified with Gram staining, catalase and oxidase test or using MALDI-TOF, while the identification at the species level was conducted through API systems (BioMèrieux). Antimicrobial susceptibility tests were performed on bacteria isolated from pure cultures, according to disk diffusion method of the European Committee on Antimicrobial Susceptibility (EUCAST version 5.0, January 2015) or standards established by the Clinical Laboratory Standards Institute using broth microdilution, and results were interpreted by consulting the breakpoint tables for MICs and zone diameters (EUCAST, Version 6.0, 2016). At UC Davis a further aliquot was subjected to culture for *Mycoplasma* spp., while an aliquot of sterile BAL from cats from Italy was examined by a conventional PCR assay targeting 280 bp of 16S rRNA gene of *Mycoplasma* spp. (14).

### Statistical Analysis

Data were analyzed and compared using Mann-Whitney (i.e., age) or Fisher's exact tests (i.e., bronchoscopy and BAL cytology findings). A *p*-value <0.05 was considered significative.

## Results

Cats in group Aa included 14 females and 10 males with a mean age of 33 ± 16 months, while cats in group FBDs included 7 males and 5 females with a mean age of 65 ± 56 months. All study cats were seronegative for FIV antibody and/or FeLV antigen.

In the group Aa, the median value of LPG was 97.5 (minimum 15.0–maximum 180.0).

### Direct Endoscopic Visualization

**Figure 1:**
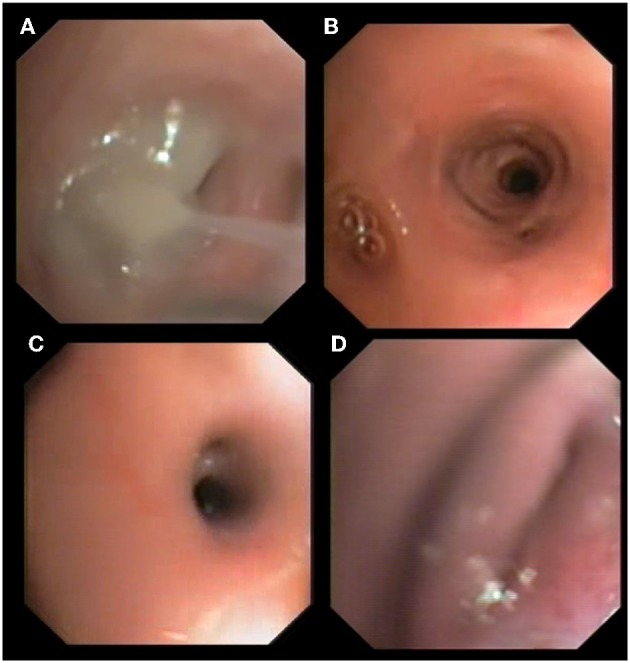
Bronchoscopic findings in cats with aelurostrongylosis. Mucus plug and bronchial obstruction **(A)**, hyperemia and stenosis **(B,C)**, and bronchial collapse **(D)**.

**Figure 2:**
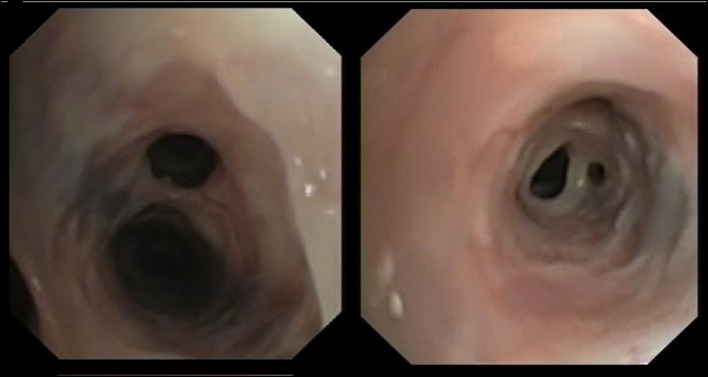
Bronchiectasis and mucus accumulation in two cats infected by *Aelurostrongylus abstrusus*.

No differences were found between Groups Aa and FBDs for the presence of bronchial mucus (79.2 vs. 91.7%; *p* = 0.64), nodular lesions (4.2 vs. 25.0%; *p* = 0.09), or airway collapse (8.3 vs. 8.3%; *p* = 1). Airway hyperemia (33.3 vs. 83.3%; *p* < 0.05), epithelial irregularities (16.7 vs. 75.0%; *p* < 0.05), and airway stenosis (12.5 vs. 66.7%; *p* < 0.05) were observed significantly more often in cats from the FBDs Group than in the Aa Group (Figure 1), while bronchiectasis (Figure 2) was present in 8 (33.3%) and 0 (0%) cats from Groups Aa and FBDs, respectively (*p* < 0.05).

### BAL Cytology and Bacteriology

No significant differences in BAL cytology were recorded between the two Groups (Table 1). Regardless of the diagnosis, BAL cytology revealed eosinophilic (Aa: 33.3%; FBD: 41.7%) and neutrophilic (Aa: 33.3%; FBD: 25.0%) inflammation as the most common findings. Two cats in the Aa Group had lymphocytic inflammation vs. one in the FBDs Group.

**Table 1:** Number (*N*) of cats with endoscopic abnormalities and bronchoalveolar lavage fluid analysis findings associated with aelurostrongylosis (Aa Group, 24 cats) and Feline Bronchial Diseases (FBD Group, 12 cats).

	**Aa group**	**FBD group**	***p*-value**
**Endoscopic finding**	***N*** **(%)**		
Excessive bronchial mucus	19 (79.2)	11 (91.7)	0.64
Airway hyperaemia	8 (33.3)	10 (83.3)	** <0.05**
Nodular lesions	1 (4.2)	3 (25.0)	0.09
Epithelial irregularities	4 (16.7)	9 (75.0)	** <0.05**
Airway collapse	2 (8.3)	1 (8.3)	1.00
Stenosis	3 (12.5)	8 (66.7)	** <0.05**
Bronchiectasis	8 (33.3)	0 (0.0)	** <0.05**
**BAL fluid**			
Neutrophilic inflammation	8 (33.3)	3 (25.0)	0.71
Eosinophilic inflammation	8 (33.3)	5 (41.7)	0.72
Lymphocytic inflammation	2 (8.3)	1 (8.33)	1.00
Mixed neutrophilic and eosinophilic inflammation	1 (4.2)	3 (25.0)	0.98
BAL cytology within normal limits	5 (20.8)	0 (0.0)	0.14
Evidence of bacteria	7 (29.2)	–	–
*Aelurostrongylus abstrusus* larvae	5 (20.8)	–	–

*p-values are shown (Fisher's exact test) with bold text indicating a statistically significant difference (p <0.05)*.

*Aa, Aelurostrongylus abstrusus; FBD, Feline Bronchial Diseases; BAL, bronchoalveolar lavage*.

One cat in the Aa Group showed neutrophilic inflammation and signs of septic inflammation (i.e., leukophagocytosis and the presence of intra- and extra-cellular bacteria), though bacterial cultures were negative. Moreover, in Aa Group cats, a multi-resistant strain of *Bordetella bronchiseptica* was cultured in 1 cat with eosinophilic inflammation, and 5 cats were PCR-positive for *Mycoplasma felis*. However, no cytological signs of sepsis were found in any of these cats.

**Figure 3:**
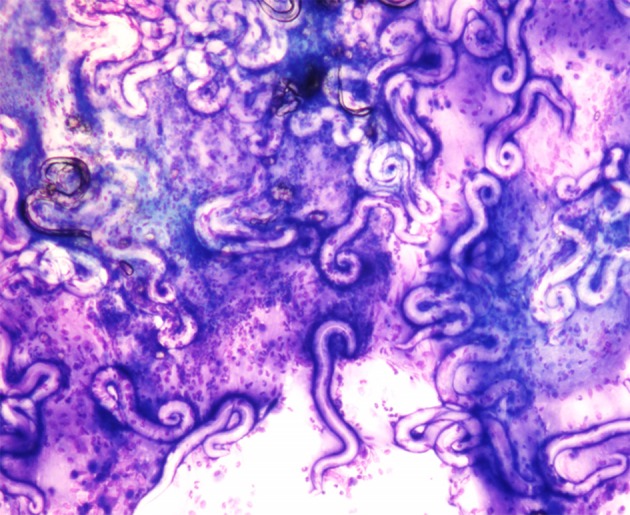
*Aelurostrongylus abstrusus* larvae from bronchoalveolar lavage cytology. Diff-Quick stain (40X).

Of the 24 *A. abstrusus* positive cats, 5 (20.8%) were positive for L1 in BAL fluid cytology (Figure 3).

## Discussion

Although bronchoscopy is one of the most useful techniques for providing a diagnosis in small animals with airway or lung diseases, the present study indicates that many visual and cytologic findings in cats infected with *A. abstrusus* and those with FBDs can overlap. Indeed, lesions visualized during lower airway endoscopy in cats with idiopathic FBD (spontaneous inflammatory airway disease, i.e., bronchitis/asthma), pneumonia or pulmonary neoplasia (adenocarcinoma and squamous cell carcinoma) did not differ with the underlying cause (11), suggesting a lack of specificity of these bronchoscopic abnormalities in cats with lower respiratory tract diseases of various etiology. This study indicates that infection with lungworms results in a similar finding. For example, the excessive mucus accumulation, a well-known protective mechanism in response of a variety of stimuli, was the most common finding in cats examined here belonging to both study groups.

Airway collapse was observed in 8.3% of cats in this study, with the same percentage affecting cats in both groups, regardless of the diagnosis. Traditionally, this is considered an uncommon finding in cats, although, in a recent study, it was visualized in 23 (48% of the study population) cats with bronchitis/asthma, neoplasia or pneumonia (11).

On the other hand, airway hyperemia and epithelial irregularities were overrepresented in cats affected by idiopathic bronchial disease in comparison to those with aelurostrongylosis. This finding was unexpected because this is observed in several conditions such inflammatory, neoplastic, and infectious disorders of the lower respiratory tract of the cat (11), and the cause and the diagnostic implications should be further investigated.

Alterations of bronchial diameter (i.e., stenosis and bronchiectasis) are usually considered uncommon in cats (11), though they were detected here with relatively high percentages in both Aa and FBDs Groups. Bronchial stenosis is generally ascribed to airway remodeling associated with feline chronic bronchitis/asthma (15, 16) and a reduction of bronchial diameter was recorded here in the vast majority of FBDs cats, whilst being almost absent in those affected by aelurostrongylosis. As suggested in a previous study, stenosis could be caused by airway remodeling because of epithelial edema, bronchial smooth muscle thickening, or by connective tissue deposition associated with chronic bronchial insult (11).

Interestingly, bronchiectasis was found in approximatively one-third of cats with aelurostrongylosis, but was not identified in any of the cats with FBDs. Moreover, although bronchial dilation predominantly affects older cat (17), cats with aelurostrongylosis and concurrent bronchiectasis examined here aged from 14 to 65 months. Bronchiectasis is reportedly an uncommon sequela of chronic inflammatory bronchopulmonary diseases, especially chronic bronchitis, neoplasia, and bronchopneumonia (11, 17) but was found frequently in cats with parasitic disease examined here.

BAL cytology represents the gold standard for non-invasive categorization of lower respiratory tract diseases in companion animals (18, 19). The present data suggest that cytologic characteristics can overlap between inflammatory and parasitic disease in cats, thus *A. abstrusus* should be included in differential diagnosis even in presence of a neutrophilic, eosinophilic and/or lymphocytic inflammation or with a BAL cytology within normal limits.

In this regard, the observation of a normal BAL cytology in 20.8% of cats with aelurostrongylosis could reflect limitations of this technique to detect abnormal cell distribution in spontaneous lower respiratory tract disease (13, 19) and analysis of different BAL sites and/or tracheobronchial brush cytology could enhance the documentation of airway inflammation in cases of *A. abstrusus* infection.

Furthermore, although lungworms and, in general, endoparasites, are expected to cause eosinophilic inflammation, only 33.3% of cats with aelurostrongylosis had such an alteration. It is possible that the eosinophilic airway response is related to the endogenous cycle of *A. abstrusus* and subsequently with the timing of sampling in relation to the course of disease, as previously suggested for peripheral eosinophilia (4).

BAL cytology does not represent a useful diagnostic tool for aelurostrongylosis, as it allowed an aetiological diagnosis in only a 20.8% of cases examined here. In fact, in a previous study, *post mortem* cytologic examination of BAL fluid showed a sensitivity of only 36.4% in detecting *A. abstrusus* larvae (20). Considering also that bronchoscopy requires anesthesia and is mildly invasive, the copromicroscopic examination should always be considered as a first step in the diagnostic work-up for feline lower airway diseases, especially in at-risk subjects living in endemic areas (4). The Baermann test is the most useful procedure to detect *A. abstrusus* larvae (3), despite some intrinsic limitations. It requires a minimum of 24 h and sometimes repeated examinations. Also, discrimination between L1s of *A. abstrusus* and those of other metastrongyloids requires careful examination and specific skills (3).

The role of bacterial co-infections in the clinical presentation of aelurostrongylosis requires further investigation. Infection was suspected in 7/24 cats in the current study based on cytologic, culture, or molecular diagnostics. Co-infection by *A. abstrusus* and *Salmonella typhimurium* was reported in a 14-week-old kitten in Australia (21). As *Salmonella* spp. typically live in animal and human intestines and respiratory salmonellosis is rare, it was proposed that a carrier state had developed during migration of *A. abstrusus* from the gastro-intestinal tract. Alternatively, *A. abstrusus* might have activated *Salmonella* bacteremia with seeding of the lung (21). It is possible that the insult elicited by the parasites resulted in entrapment of microbes in the bronchial mucus secondary to a dysfunction of the muco-ciliary elevator, with the onset of the secondary infection.

*Bordetella* colonizes the respiratory tract of small animals and is not usually isolated from other tissues. It is considered a commensal, though it can act as a primary pathogen of cat respiratory tract. Overcrowding and concurrent viral infections, e.g., feline calicivirus and herpesvirus, can predispose cats to infection and disease (22). Hence, it could be hypothesized that lungworms may have created the conditions for the bacterial overgrowth in the cat examined here.

Despite the absence of microbial growth, septic neutrophilic inflammation observed in BAL fluid cytology from another cat of Group Aa could be considered a reliable indicator of bacterial infection, indicating that underlying parasitic pneumonia should be considered as a risk factor for bacterial pneumonia. In a recent study, no microbial growth was observed in three dogs with cytological evidence of bacteria (6), indicating that both culture and cytology should be used to determine the presence of infection.

*Mycoplasma felis* is the most commonly reported mycoplasma in the feline lower respiratory tract (1) and, although its role in lower respiratory tract infection is debated, its detection could represent a true infection. In some studies, *Mycoplasma* species are thought to act as a primary pathogen in lower respiratory disease in cat (23–25). Although the potential for oropharyngeal contamination during sampling must be considered, *A. abstrusus* may create predisposing conditions for mycoplasmal colonization and proliferation through an impairment of local defense mechanisms. Further studies are warranted to establish the role of *Mycoplasma* co-infections in feline lungworm disease.

There are some limitations inherent to the clinical aspects of this study. The visualized abnormalities described were subjective and based only on the experience of the endoscopist. One clinician performed all the endoscopies in the US (LRJ) while another clinician performed all the endoscopies in Italy (PEC), however all findings were agreed upon in advance and interpreted using a detailed procedure description as an interpretation guide (11).

## Conclusions

Bronchial alterations in aelurostrongylosis are indistinguishable from those observed in other feline airway diseases of inflammatory etiology and thus also infectious or neoplastic causes. Therefore, it can be argued that some bronchial lesions are similar in nature, regardless of the cause of the disease, including lungworms. Bronchoscopy in cats with aelurostrongylosis, although not of high diagnostic efficiency in the clinical setting, may represent a useful ancillary tool to evaluate the presence, extent and type of bronchial abnormalities. Furthermore, it allows the identification of irreversible lesions, e.g., bronchiectasis or stenosis, that could potentially affect long-term prognosis. Finally, although prompt anthelminthic therapy against *A. abstrusus* usually provides a rapid and complete clinical recovery (3), some cats need repeated administrations, and clinical and radiographic signs can persist for several weeks (4). In these cases, as much information as possible is required, and a careful clinical evaluation, including bronchoscopy, BAL cytology, and bacterial culture, could be helpful in identifying the most appropriate supportive therapy.

## Data Availability Statement

The datasets generated for this study are available on request to the corresponding author.

## Ethics Statement

The animal study was reviewed and approved by Committee on Animal Research and Ethics at the Universities of Chieti-Pescara, Teramo and Experimental Zooprophylactic Institute of AeM (CEISA). Written informed consent was obtained from the owners for the participation of their animals in this study.

## Author Contributions

PC, LJ, MD, and SP carried out the clinical procedures. AD and SM carried out all parasitological procedures. AB, AD, FP, and RS supervised the project. All authors conceived and planned the experiments and contributed to the interpretation of the results and to the final manuscript.

### Conflict of Interest

FP and RS were employed by company Bayer Animal Health. The remaining authors declare that the research was conducted in the absence of any commercial or financial relationships that could be construed as a potential conflict of interest.
